# Supplementary material for: Swift Realisation of Wastewater-Based SARS-CoV-2 Surveillance for Aircraft and Airports: Challenges from Sampling to Variant Detection
Source: Microorganisms. 2025 Aug 8;13(8):1856. doi: 10.3390/microorganisms13081856 (PMC12388640; doi:10.3390/microorganisms13081856)
Supplement: Supplementary file 1 [file microorganisms-13-01856-s001.zip › Airport_SI.pdf]

microorganisms

MDPI

Supplementary Information

Swift Realisation of Wastewater-Based SARS-CoV-2 Surveillance for Aircraft & Airports: Challenges from Sampling to Variant Detection

Natalie Marquar[1†], Cristina J. Saravia[2†*], Kira Zachmann[3†], Ulrike Braun[1], Claus Gerhard Bannick[2], Timo Greiner[4], Peter Pütz[4], Susanne Lackner[3], Shelesh Agrawal[3]

[1] Wastewater Analysis, Monitoring Methods, German Environment Agency, Berlin, Germany
[2] Wastewater Treatment Technology, Wastewater Disposal, German Environment Agency, Berlin, Germany
[3] Chair of Water and Environmental Biotechnology, Institute IWAR, Department of Civil and Environmental Engineering Sciences, Technical University Darmstadt, Darmstadt, Germany
[4] Department of Infectious Disease Epidemiology, Robert Koch Institute, Berlin, Germany
[†] Authors contributed to the manuscript equally
[*] Correspondence: cristina.saraviaarzabe@uba.de

1. Materials and Methods

1.1 ddPCR analysis

Droplet Digital PCR (ddPCR) from Bio-Rad was used to quantify the genes N2 and E of SARS-CoV-2 in the airport samples, after the extraction of viral nucleic acids using the Wizard Enviro Total Nucleic Acid Kit (Promega). The samples were treated according to the protocol provided by the manufacturer of the PREvalence ddPCR SARS-CoV-2 Wastewater Quantification Kit (Bio-Rad). 9.9 µl aliquots of the extracted nucleic acids were mixed with 12.1 µl of the master mix (Bio-Rad ddPCR Supermix, primers and probes dye) to enable the processing of 20 µl per sample and 70 µl of Droplet Generation Oil in the cartridge. This cartridge was then transferred to the QX200 Droplet Generator, which uses microfluidics to randomly create droplets by combining oil and sample. A no template control (NTC, nuclease-free water) and a positive reverse transcription ddPCR control were included in every run. After PCR (see thermal cycle profile below), the droplets were stabilised for 45 minutes in the refrigerator before being counted in the Bio-Rad QX200 Droplet Reader (fluorescence channels FAM and HEX). The data were analysed using the QX Manager Software Standard Edition, Version 1.2, immediately after plate reading was complete. To minimise potential contamination, extraction of viral nucleic acids and ddPCR were performed in separate laboratories.

Table S1: Thermal cycle profile – PREvalence ddPCR SARS-CoV-2 Wastewater Quantification Kit

Step	Description	Temperature, °C	Time	Cycles
1	Reverse transcription	50	60 min	1
2	Reverse transcriptase deactivation	95	10 min	1
3	Denaturation	94	30 sec	40
4	Annealing/extension	55	1 min	
5	Enzyme deactivation	98	10 min	1
6	Droplet stabilization	4	30 min	1
7	Hold	4	∞	1

1.2 dPCR analysis

For the quantification of SARS-CoV-2 in wastewater samples, dPCR was performed using the QuantStudio Absolute Q Digital PCR System (Thermo Fisher) and two different kits according to the manufacturer's instructions: The Absolute Q dPCR SARS-CoV-2 Wastewater Surveillance Kit and the QuantStudio Absolute Q M16 Digital PCR Kit. 9µl of total master mix was prepared for each sample and was loaded in the QuantStudio Absolute Q MAP16 Digital PCR Plate (Thermo Fisher Scientific). A total of 7 µL of RNA was used for each reaction. A negative control was included in each PCR run as a quality control measure. FAM, HEX, and ROX fluorescent dyes were employed in this study. The dPCR protocol involved an initial reverse transcription step and is shown in Table S2. The primers used in the dPCR specifically targeted the N1 and N2 gene markers of the N-protein gene. The data was analysed using the QuantStudio Absolute Q Digital PCR Software.

Table S2: Thermal cycle profile – Absolute Q dPCR SARS-CoV-2 Wastewater Surveillance Kit

Step	Description	Temperature, °C	Time	Cycles
1	Reverse transcription	50	10 min	1
2	Initial denaturation	96	5 min	1
3	Denaturation	96	5 sec	45
4	Annealing / Extension	62	30 sec	45

2. Results

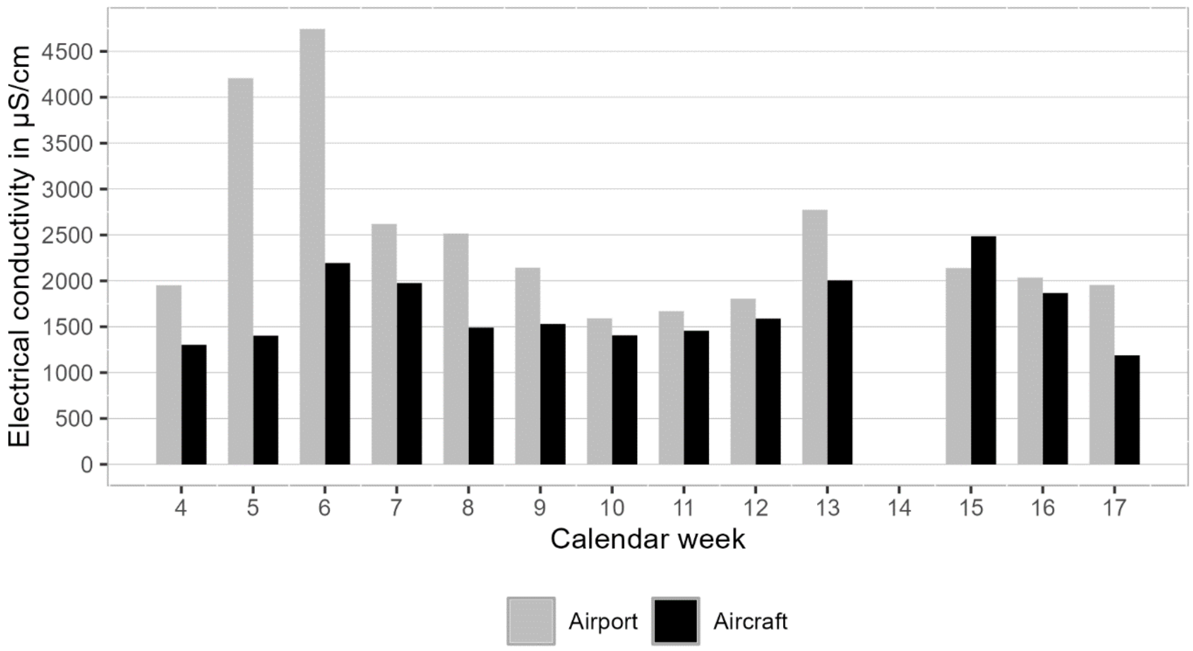

Figure S1: Electrical conductivity for the aircraft and airport samples for all sampling days

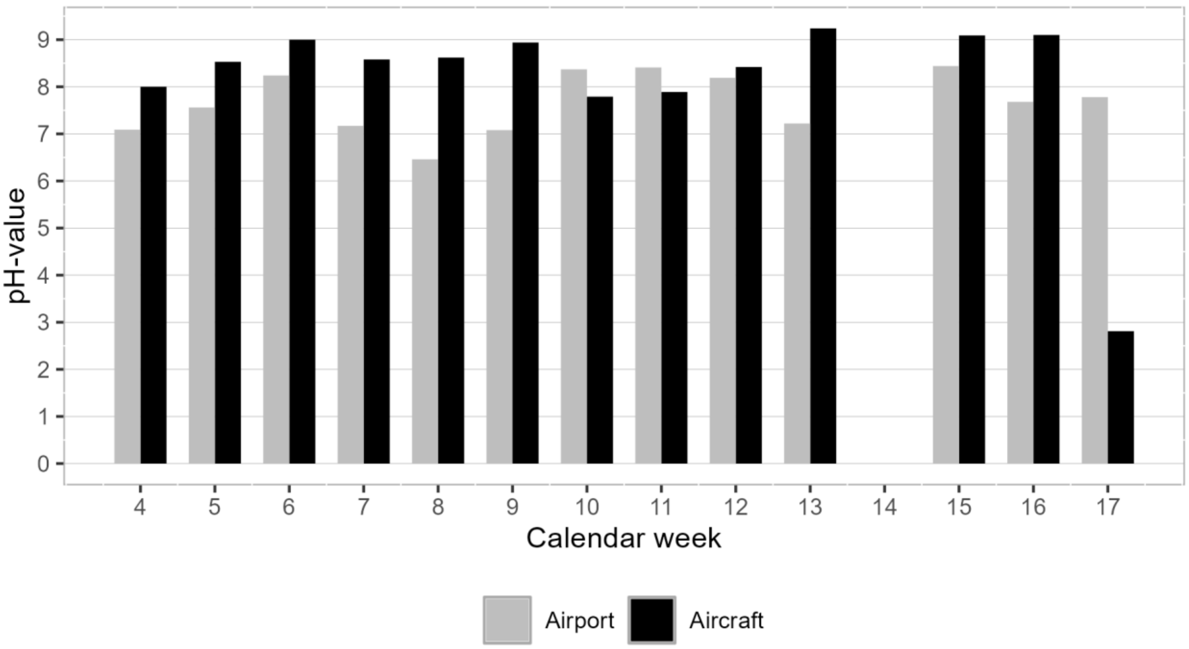

Figure S2: pH-value for the aircraft and airport samples for all sampling days

Table S3: SARS-CoV-2 concentration in airport wastewater samples measured by ddPCR

Date	SARS-CoV-2 concentration in gene copies/mL wastewater	
	E	N2
27.01.2023	68.62	103.11
03.02.2023	79.11	108.44
10.02.2023	60.62	79.91
17.02.2023	438.22	547.56
24.02.2023	737.54	1203.60
03.03.2023	1142.22	1286.67
10.03.2023	242.22	408.89
17.03.2023	120.67	86.22
24.03.2023	848.89	1053.33
31.03.2023	392.89	586.67
14.04.2023	134.89	210
21.04.2023	200	281.78
28.04.2023	142.22	254.67

Table S4: SARS-CoV-2 concentration in airport and aircraft wastewater samples measured by dPCR

Location	Date	SARS-CoV-2 concentration in copies/mL wastewater
Airport	27.01.2023	7.85E+04
Aircraft	27.01.2023	1.93E+03
Airport	03.02.2023	6.27E+05
Aircraft	03.02.2023	3.85E+03
Airport	10.02.2023	1.87E+05
Aircraft	10.02.2023	1.58E+04
Airport	17.02.2023	3.03E+05
Aircraft	17.02.2023	3.85E+03
Airport	24.02.2023	7.23E+05
Aircraft	24.02.2023	1.96E+04
Airport	03.03.2023	8.49E+05
Aircraft	03.03.2023	1.43E+05
Airport	10.03.2023	1.34E+06
Aircraft	10.03.2023	2.09E+05
Aircraft	17.03.2023	7.70E+03
Airport	17.03.2023	1.84E+05
Airport	24.03.2023	1.09E+04
Aircraft	24.03.2023	1.51E+06
Airport	31.03.2023	1.49E+05
Aircraft	31.03.2023	2.35E+05
Airport	31.03.2023	8.65E+05
Airport	14.04.2023	5.50E+05
Aircraft	14.04.2023	1.83E+05
Airport	21.04.2023	1.17E+06
Aircraft	21.04.2023	8.72E+04
Airport	28.04.2023	8.69E+05
Aircraft	28.04.2023	0
